# Eliciting preferences in glaucoma management—a systematic review of stated-preference studies

**DOI:** 10.1038/s41433-023-02482-3

**Published:** 2023-03-21

**Authors:** L. M. J. Scheres, M. Hiligsmann, L. van Gorcom, B. A. B. Essers, H. J. M. Beckers

**Affiliations:** 1 University Eye Clinic Maastricht, Maastricht University Medical Centre+, Maastricht, the Netherlands; 2 Department of Health Services Research, Care and Public Health Research Institute (CAPHRI), Maastricht University, Maastricht, the Netherlands; 3 Department of Clinical Epidemiology and Medical Technology Assessment, Maastricht University Medical Centre+, Maastricht, the Netherlands

**Keywords:** Glaucoma, Surgery, Health services

## Abstract

**Background:**

In most cases, glaucoma patients require long-term medical and/or surgical treatment. Preference studies investigate how different aspects of glaucoma management, such as health or process outcomes, are valued and herewith help stakeholders make care more responsive to patients’ needs. As, to our knowledge, no overview of these studies is currently available, this study aims to systematically review and critically appraise these studies.

**Methods:**

A systematic literature review was conducted using keywords for stated-preference studies and glaucoma up to October 2021. Studies were included if they were original research and used a stated-preference methodology to investigate preferences in patients or healthcare professionals for different aspects of glaucoma management. Data were extracted and summarized. Furthermore, a quality appraisal of the included studies was performed using two validated checklists.

**Results:**

The search yielded 1214 articles after removal of duplicates. Of those, 11 studies fulfilled the inclusion criteria. Studies aimed to elicit preferences for glaucoma treatment (27%), glaucoma related health state valuation (36%), and services (36%) from the patient (91%) or ophthalmologists’ perspective (9%). Altogether studies included 69 attributes. The majority of attributes were outcome related (62%), followed by process (32%) and cost attributes (6%). Outcome attributes (e.g., effectiveness) were most often of highest importance to the population.

**Conclusions:**

This systematic review provides an up-to-date and critical review of stated-preference studies in the field of glaucoma, suggesting that patients have preferences and are willing to trade-off between characteristics, and revealed that outcome attributes are the most influential characteristics of glaucoma management.

## Abstract (zh)

**背景:**

大多数情况下, 青光眼患者需要长期治疗和/或手术治疗。偏好研究通过评估青光眼治疗的不同方面, 例如治愈或是过程结局, 帮助相关人员在诊疗中更符合患者的需求。据我们所知, 目前还没有关于这些研究的综述, 本研究旨在系统地回顾和批判性地评价这些研究。

**方法:**

使用陈述性偏好研究和青光眼作为关键词, 对截至2021年10月的文献进行了系统回顾。纳入标准为原创性的, 并且使用陈述偏好的方法来调查患者或医护人员对青光眼诊疗不同方面的偏好研究。我们对数据进行了提取和总结, 并使用两个有效的检查表对纳入的研究进行了质量评估。

**结果:**

在去除相同的文章后, 搜索得到了1,214篇文章。其中, 11项研究符合纳入标准。研究旨在从患者 (91%) 或眼科医生 (9%) 的角度, 征求其对青光眼治疗 (27%) 、青光眼相关健康状态评估 (36%) 和服务 (36%) 的偏好。研究共包括69个属性。大多数属性与结局有关 (62%), 其次是过程 (32%) 和成本属性 (6%) 。结局属性 (如疗效) 对人群来说往往是最重要的。

**结论:**

本系统综述对青光眼领域的陈述性偏好研究进行了最新的批判性回顾, 表明患者有偏好, 愿意在各种特征之间进行权衡, 并揭示了结局属性是青光眼管理中最具影响力的特征。

## Introduction

Glaucoma is the leading cause of irreversible and preventable blindness in the world [1]. The disease represents a group of chronic progressive neuropathies characterized by structural damage to the optic nerve, leading to visual field defects. Currently, the only known treatment is lowering intraocular pressure (IOP) to prohibit further damage [2]. In order to lower IOP, various therapies are available, of which the most frequently used is pharmaceutical therapy. Filtering surgery is only considered when topical therapies and laser treatment have failed and is generally regarded as the treatment of last resort by both patients and healthcare professionals [3, 4]. During the last decade, novel less and minimally invasive glaucoma surgeries (MIGS) have revolutionized glaucoma treatment [5]. These MIGS procedures are designed to lower IOP with a high safety profile, a quick postoperative recovery, and can be used in earlier disease stages. However, recent studies show that IOP reduction (i.e., effectiveness) after MIGS procedures might be less favourable than with traditional filtering surgery [6].

Physicians are used to judging the success of glaucoma management by using parameters such as IOP and visual field defects. Yet, treatments do not only differ in effect on IOP, but also vary in adverse events profile, affordability, recovery time, duration of effect, and monitoring burden. Patients’ perspectives and preferences on these aspects of the procedures have been given less attention, even though their views are becoming increasingly important in healthcare interventions [7].

In terms of encouraging patient-centred medicine, it is crucial to determine what patients prefer, inform them, and then support them in the decision-making process [8]. The concept ‘preference’ represents the desirability or value of a health-related outcome, process, or treatment choice [9]. Preference studies help healthcare professionals to broaden their understanding of patient values. Incorporating these values into clinical and policy decisions may lead to decision-making that better reflects the preferences of stakeholders such as patients [10]. Stated-preference studies are gaining more popularity for preference elicitation and aim to explore the trade-offs participants are willing to make by constructing a hypothetical choice set in an experimental framework [11].

Preference research into the treatment of glaucoma is particularly interesting, as some treatments are associated with higher efficacy but can also lead to more side effects. Patients often have to decide whether a reduced probability of the disease worsening outweighs the risk of potential (serious) adverse events. Moreover, clinical endpoints that measure the efficacy of treatments, such as IOP, can be difficult to reflect on the patient’s perspective. As the European Glaucoma Society guidelines state “The goal of care for people with glaucoma is to promote their well-being and quality of life, and patient preferences should be taken into account” [12]. For this reason, decisions should consider all aspects that impact their daily life, including disease-related health states, the process of care, and treatment preferences.

Whilst general reviews of stated-preference studies in health care have been carried out, none of these has specifically investigated glaucoma. Given the interest in preference studies, the increased number of relevant studies, and the new opportunities and challenges of managing glaucoma, a review that focuses on glaucoma is timely. Therefore, the purpose of our systematic review is to provide an overview and synthesis of all stated-preference experiments eliciting preferences of glaucoma patients and their healthcare professionals, to assess their quality, and to determine which attributes are valued as most important.

## Methods

### Search strategy and screening

A systematic review of the literature was conducted in line with guidelines published by PRISMA (Preferred Reporting Items for Systematic Reviews and Meta-Analyses) [13]. Electronic searches were conducted in the EMBASE and PubMed databases on the 27th of October 2021 using a combination of keywords and synonyms of stated-preferences and glaucoma (see Appendix I). Search terms were derived from previously published reviews of stated-preference research in health care by de Bekker et al. [14], Clark et al. [15], and Soekhai et al. [16]. A comprehensive search strategy was used to ensure that all studies, regardless of naming, were captured. The results of the database searches were imported into an EndNote library and de-duplicated.

Two researchers independently conducted the search for relevant publications. First, titles and abstracts of all articles identified by the search strategy were screened, followed by an evaluation of full texts of residual articles. Finally, a backward and forward reference search strategy using Web of Science was performed on included studies. Any disagreements between the two researchers were discussed until a consensus was reached.

Stated-preference studies were included if they investigated patients’ or healthcare providers’ preferences for different aspects of glaucoma management and were published in full-text English. Stated-preference studies were defined as methods using ranking, rating, or choice designs to quantify preferences for various attributes [10]. There were no constraints on publication year, age, sex, or origin of the participants. Contingent valuation surveys (WTP) were excluded. Lastly, case reports and conference abstracts were also excluded.

### Data extraction

Data extraction and analysis were performed in several steps. In the first step, general characteristics and results of included studies were collected and summarized in a data extraction sheet (Appendix II). Extracted characteristics covered general study characteristics such as the first author’s last name, year of publication, country, study aim, application, and study population. The data extraction was complemented with more detailed information on the study design, including attribute and level identification and selection, instrument design, and survey administration. Included attributes were then categorised into three main categories (a) outcome, (b) process, and (c) cost [17, 18]. Outcome attributes can refer to IOP control, adverse effects, patient-reported outcomes, and the accuracy of diagnostics. The process category comprehends all characteristics related to the delivery of care, such as mode of administration and frequency of visits. If appropriate, these main categories were further classified into subcategories for attributes with shared characteristics (such as effectiveness and adverse effects for the category outcome), allowing for a more comprehensive synthesis of results. A second researcher was consulted in case of uncertainty about the attribute categorization. Finally, the relative importance of attributes was evaluated, as was previously conducted in similar reviews in health care [17–19]. If relative importance values were directly reported, these were used. Otherwise, if coefficients for attribute levels were stated, relative importance was calculated using the range method as recommended by the ISPOR (International Society for Pharmacoeconomics and Outcomes Research) Conjoint Analysis Good Research Practices Task Force [20]. This method determines the range of attribute-specific levels by calculating the difference between the lowest and the highest coefficient for the levels of each attribute. The relative importance is then calculated by dividing the range by the sum of all attribute level ranges. Studies were excluded from the relative importance analysis if no information was available on attributes’ relative importance scores or their level coefficients. For each attribute, relative importance was calculated and compared per study application (treatment, valuation of glaucoma-related health states, service). Additionally, the number of times an attribute (sub)category was identified as first or second most important was evaluated by comparing the relative importance scores of all attributes.

### Quality appraisal

In line with previous reviews, quality appraisal of the identified studies was performed using two checklists [18, 19]. For the first assessment, the PREFS checklist was used [21]. This checklist evaluates five criteria: Purpose, Respondents, Explanation, Findings, and Significance. Every criterion was assessed with either a score of 0 or 1 and summed up for each study. The PREFS checklist was complemented by the checklist published by the ISPOR Guidelines to provide a more in-depth evaluation [10]. This checklist includes ten main elements with three subquestions: research question, attributes and levels, task construction, experiment design, preference elicitation, instrument design, data collection plan, statistical analyses, results and conclusions, and study presentation. Each subquestion was appraised with a score of 1 if a study reported on at least some aspect of its criterion and a score of 0 if it did not. The sum of the score for each subquestion gives the final score for a study. The quality appraisal was performed by two researchers separately (LMJS and LvG), and discrepancies were resolved in consensus.

## Results

**Fig. 1:**
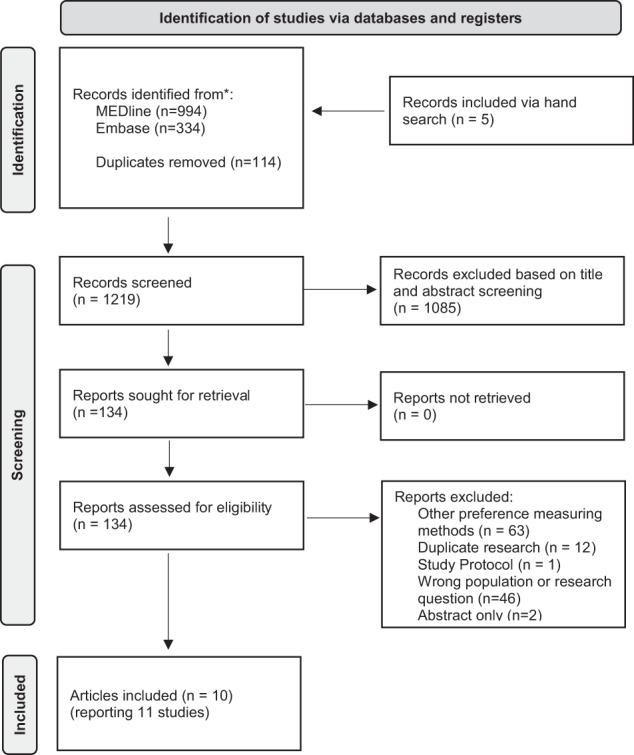
Flow diagram of study selection adapted from: The PRISMA 2020 statement: an updated guideline for reporting systematic reviews [41].

The search resulted in 994 findings through PubMed and 334 through EMBASE. A further five records were included from backward and forward citation searches, and one hundred and fourteen duplicates were removed. Ten articles presenting 11 studies met the inclusion criteria and remained for synthesis after the selection process (Fig. 1) [22–32]. One of the articles presents two separate studies [29].

### Study characteristics

Table 1 presents an overview of the study characteristics. More detailed information is shown in Appendix II. Studies were published between 2005 [24] and 2021 [29, 30]. All studies were performed in high-income countries (HIC), mainly in the UK (*n* = 5). Sample sizes ranged from 32 [29] to 500 [32] participants, with an average sample size of 173 participants per study.

**Table 1:** Descriptive study characteristics (*n* = 11).

	% (*n*)
Country	
Australia	9% (1)
Germany	18% (2)
Singapore	18% (2)
UK	45% (5)
USA	9% (1)
Year of publication	
2005	9% (1)
2006	9% (1)
2007	9% (1)
2008	18% (2)
2017	9% (1)
2019	18% (2)
2021	27% (3)
Target population	
Patients (glaucoma)	91% (10)
All types	50% (5)
Mild/moderate stage	10% (1)
No comorbidities	20% (2)
On topical medication	10% (1)
Open-angle glaucoma	10% (1)
Health care providers	9% (1)
Application	
Treatment	27% (3)
Health state valuation	36% (4)
Service	36% (4)
Number of attributes	
4	9% (1)
5	45% (5)
6	9% (1)
7	27% (3)
13	9% (1)

Studies examined preferences for three different applications. Preferences for glaucoma treatment were investigated in three studies, and these studies mainly aimed to assess preferences for benefits and risks of treatment. Preferences for glaucoma-related health state valuation were studied in four studies, investigating the perceived importance of glaucoma-related quality of life outcomes. The final four studies investigated preferences for glaucoma service and aimed to determine priorities in follow-up services, diagnostics, and delivery of care. Regarding the population, respondents were primarily glaucoma patients receiving treatment (*n* = 10). There was variability concerning participants’ treatment history (e.g., topical or surgical treatment) and disease severity. One study assessed the preferences of 41 healthcare providers (ophthalmologists) [29].

### Quality appraisal

Table 2 reports the quality assessment of the studies included. When appraised according to the PREFS checklist, most publications scored four out of five points. One study [29] scored five out of five, and two studies scored three out of five [22, 26]. Every included study reported a purpose related to the identification of preferences and significance of the study, clearly explained the methods, and presented statistical analyses to support the results. However, reporting on examining the differences between responders and non-responders of the respondent sample was lacking in all studies except for one [29].

**Table 2:** Quality appraisal of stated-preference studies regarding glaucoma management.

Study	Aspinall et al. (2005)	Aspinall et al. (2008)	Bhargava et al. (2006)	Bhargava et al. (2008)	Burr et al. (2007)	Fenwick et al. (2021)	Le et al. (2019)	Lu et al. (2019)	Muth et al. (2021)	Ozdemir et al. (2017)	Total
PREFS Checklist Joy et al. (2013)											
Purpose	1	1	1	1	1	1	1	1	1	1	100%
Respondents	0	0	0	0	0	0	0	0	1	0	10%
Explanation	1	1	1	1	1	1	1	1	1	1	100%
Findings	1	1	1	0	0	1	1	1	1	1	80%
Significance	1	1	1	1	1	1	1	1	1	1	100%
Total score	4	4	4	3	3	4	4	4	5	4	78%
ISPOR Checklist Bridges et al. (2011)											
Research question	2	3	3	3	3	3	3	3	3	3	97%
Attributes and levels	3	2	2	3	3	3	3	3	1	3	90%
Construction of task	3	3	3	3	3	3	3	3	3	3	100%
Experiment design	3	3	3	3	3	3	3	3	2	3	97%
Preference elicitation	2	2	1	3	3	3	3	2	2	2	77%
Instrument design	2	2	1	2	3	3	3	3	2	2	77%
Data collection plan	3	3	2	2	2	3	2	3	2	2	80%
Statistical analyses	1	1	2	2	2	2	1	2	2	1	53%
Results and conclusions	2	2	2	3	3	3	3	3	2	3	87%
Study presentation	2	3	3	2	3	3	3	3	3	3	93%
Total score	23	24	22	26	28	29	27	28	22	25	85%

Regarding the ISPOR checklist, quality scores varied between 22 [23, 29] and 29 [28] out of 30, with a median score of 25. Most items were described in detail, though items most frequently lacking were item 6 (instrument design), item 7 (data collection), and item 8 (statistical analyses).

Methods used for attribute and level identification and selection were most often expert consultation (81%), followed by literature review (55%), qualitative interviews (36%), and focus groups (27%) (Table 3). Studies often used a combination of methods and did not distinguish between level identification and selection. Regarding data collection, only four studies justified the choice of sample size [24, 25, 28, 30]. Ozdemir et al. did not provide a sampling strategy but a target number of respondents of 500 [32].

**Table 3:** Methods used for study design of stated-preference research regarding glaucoma management.

	% (*n*)
Attribute and level identification	
Expert consultation	81% (9)
Literature search	55% (6)
Qualitative patient interviews	36% (4)
Focus groups	27% (3)
Preliminary research	27% (3)
Attribute and level selection	
Expert consultation	73% (8)
Literature search	55% (6)
Qualitative patient interviews	36% (4)
Focus groups	27% (3)
Mode of administration	
Interview administered interview	73% (8)
Online or paper survey	27% (3)

Survey length varied considerably, with a total number of tasks ranging from 10 to 36. A ‘block’ design was often used to limit the number of tasks. None of the studies added an opt-out option in the choice task. Few studies undertook pilot testing to improve instrument design, confirm respondents understanding of the task, and test the level of burden of the instrument. Most studies used interviewer-administered surveys (73%), and three used an online or paper self-administered questionnaire [26, 27, 32].

Regarding the item ‘statistical analyses’, the subitems ‘assessment of respondent characteristics’ and ‘quality of the responses’ were often underreported. While respondent characteristics were reported in most of the studies, none of the studies compared these with the desired population. Furthermore, testing the quality of responses by evaluating the internal validity of the data was lacking in four studies [24, 25, 27, 32]. Nevertheless, all but two studies did not report on sensitivity analysis [26, 27]. No serious conflicts of interest were reported.

### Attributes

The average number of attributes was 6.3 (ranging from 4 to 13), while the average number of levels used in the studies was 3.0 (ranging from 2 to 5). Of the 69 reported attributes, 43 were classified as outcome attributes (62%), 22 as process attributes (32%), and four as cost attributes (6%). Outcome and process attributes were diversely framed depending on the study’s aim and setting. These categories were further divided into subcategories. The complete list of attributes, their levels, and the corresponding studies is included in Appendix III. In most studies, many attributes were significant and herewith of importance in decision-making.

Twenty-seven of the 43 outcome attributes (63%) were related to quality of life [24–27, 30], which was, therefore, the most commonly considered subcategory. Effectiveness attributes (*n* = 9) were included in five studies [23, 27, 29, 30]. Three studies included attributes related to adverse effects [23, 27, 30]. The most common included adverse effect was ‘eye discomfort’ (*n* = 3).

Process attributes were included in six studies [22, 23, 28, 29, 32] and were subcategorised in mode of administration, frequency, location, and waiting times. The most frequently included process attribute was mode of administration which was included in six studies [22, 23, 28, 29, 32]. The attribute subcategory mode of administration was framed variously from ‘preference for a trabeculectomy or topical therapy’ [23] to ‘comfort’ [29] and ‘expertise’ [22, 28].

Four studies included cost attributes [28, 29, 32], either ‘costs per time’ [28, 29] or ‘yearly costs’ [32].

### Relative importance

Of the 11 studies, one was excluded from the relative importance analysis since it provided preference weight graphs but no attribute weights nor level coefficients [24]. Hence, data from ten studies were used. Appendix IV of the supplementary material provides an overview of relative importance scores of attributes per study. In one study, attribute levels were interacted with a survival attribute which was accounted for in the relative importance calculation [30].

**Fig. 2:**
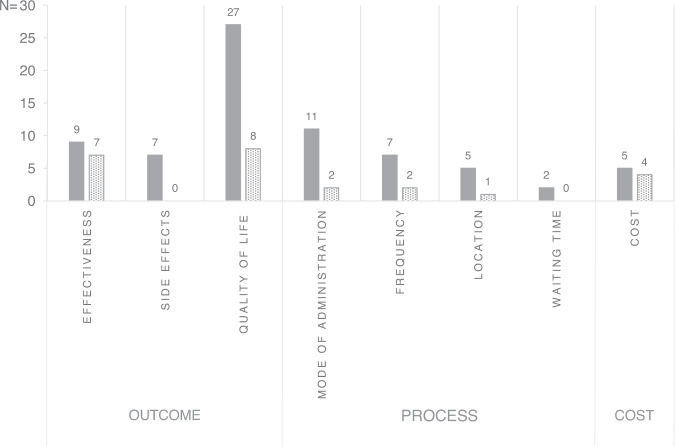
Number of times an attribute (sub)category was used and valued as first or second most important.

The relative importance scores of attributes were compared, and the attribute with the highest relative importance score within a study was regarded as the most important. Figure 2 shows how often a subcategory of attributes was included and the number of times a subcategory was considered first or second most important. Outcome attributes, particularly the subcategory effectiveness, were ranked most often as the most important relative to the total number of included attributes of the (sub)category. Outcome attributes were considered most important in 8 out of 12 analyses (effectiveness five times and quality of life outcomes three times), followed by process attributes in four analyses (frequency two times, mode of administration and location each one time). Moreover, process attributes were only considered the most important when outcome attributes were not included.

**Fig. 3:**
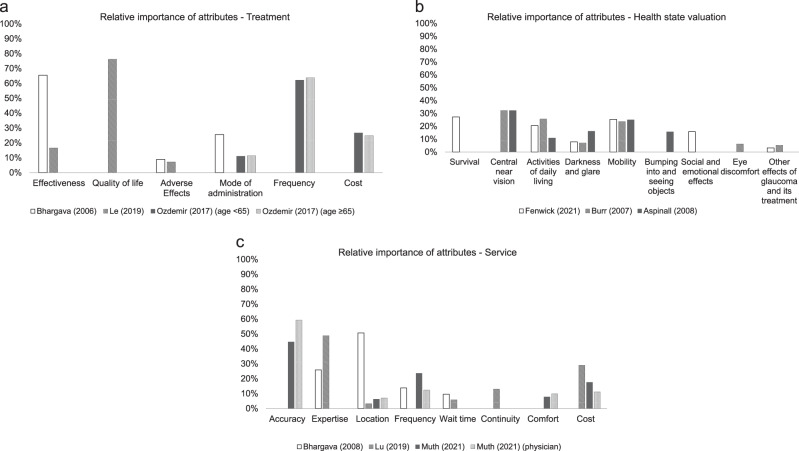
Relative importance of attributes specified per study application for treatment (**a**), valuation of glaucoma-related health states (**b**), and service (**c**) studies.

Figure 3 presents the relative importance of attributes specified per study application for treatment, valuation of glaucoma-related health states, and service studies, respectively.

#### Treatment

Preferences for glaucoma treatment were evaluated in three studies. The framing of the attributes varied substantially between studies. If an effectiveness outcome attribute was included in a study, it was considered the most important attribute. The most important attributes were ‘ability to drive’ [23, 27] and ‘having control of intraocular pressure’ [27]. In the study of Bhargava et al., the risk of moderate visual loss (the ability to drive) was valued as more important than the risk of long-term blindness [23]. When an adverse effects attribute was considered, it was deemed less important than effectiveness [23, 27]. One study included a cost attribute [32], which was of the second highest importance in both subgroup analyses (age <65 years and age ≥65 years) in comparison to the (process) attribute ‘frequency’ which was of the highest importance. No outcome attributes were included in this study.

#### Health state valuation

Valuation of glaucoma-related health states was reported in four studies. One study reported preference utility weight graphs but no values [24]. As is expected for this application, studies only included outcome attributes. Central and near vision was identified as the most important attribute in three studies [24–26], with relative importance values ranging from 32.2% [26] to 32.3% [25]. The fourth study did not include this attribute [30]. ‘Outdoor mobility’, including the ability to drive, and ‘activities of daily living’ were the second most important attributes in all studies. Adverse effects such as ‘eye discomfort’ and ‘other (systemic) adverse effects of treatment’ were included in two studies and found to be of the least importance (ranged 0.0% to 6.2%) [26, 30].

#### Service

Glaucoma service was reported in four studies. If outcome attributes were included (*n* = 2), these were of the highest importance for patients as well as physicians [29]. Regarding the studies that included process and cost attributes only, ‘expertise of the healthcare professional’ was of the first or second highest importance (range 25.9% to 48.9%) [22, 28]. The attributes that were valued as least important by patients were ‘waiting time’ (range 5.8% to 9.6%) [22, 28] and ‘location’ (3.2% to 6.3%) [28, 29]. This finding is in contrast with the results of the study by Bhargava et al., where travel time was valued as the highest importance (35.4%) [22].

## Discussion

This systematic review identified 11 stated-preference studies in glaucoma management. Studies were conducted to assess preferences in three applications: glaucoma treatment, glaucoma-related health status valuation, and glaucoma services. In each type of application, respondents were found to be willing to trade-off, which makes stated-preference techniques suitable. The majority of studies assessed patients’ preferences, although one study assessed the preferences of physicians. In most studies, the included attributes were significant and, therefore, important in clinical decision-making.

Overall, outcome attributes were most often included in the experiments and considered the most prominent in terms of relative importance. In some studies, process and cost attributes were significant predictors of preference as well. Even though a few studies have evaluated cost attributes, these are difficult to interpret across countries when they have varying healthcare systems and reimbursement schemes. This distinction may then render results less generalizable.

Concerning treatment studies, the effectiveness of treatment was shown to be the most influential characteristic, while adverse effects were deemed of lesser importance. Interestingly, adverse effects were also considered the least important in the health state valuation studies. The finding that adverse effects are of lesser importance is unexpected, given the burden of topical medication on ocular comfort. An explanation could be the framing of the attributes and their levels. The attributes related to adverse effects were framed relatively mild and tended to concern ocular comfort, but no serious adverse effects. Another possible explanation for this finding could be that the respondents were still in the early stages of glaucoma and had not been exposed to the burden of treatment. Patients with a combination of three or more medications tend to experience higher levels of ocular symptoms [33].

Furthermore, in the studies investigating glaucoma-related health status valuation, central vision was valued as the most important to patients, even though glaucoma is characterized by peripheral vision loss. This alongside outdoor mobility, including the ability to drive, which was often valued as the second most important. The ability to drive is a commonly identified functional limitation of patients with glaucoma and is considered a key feature of independence [34–36].

In studies that aimed at prioritizing aspects of glaucoma service, efficacy of diagnostics and expertise of health care professional were of the highest importance. Contradictory, travel time (process subcategory: location) was found to be of the highest importance in the study of Bhargava et al. [22]. Yet, it was found to be of lowest importance in Lu et al. [28], even though similar levels were used. This difference might be due to the fact that the study by Lu et al. was conducted in Australia, where people are used to longer travel times, while that of Bhargava was conducted in the UK, which emphasizes the impact of circumstances and perspective of responders when drawing conclusions from preference research.

Discrepancies in relative importance between similar attributes were seen more often because the studies were rather heterogeneous in terms of aim, (framing of) attributes and their levels, and the methods used. The selected attributes, and therewith the preferences elicited, depend on the specific research question. Different research questions may have led to different selection and framing of attributes and their levels and, consequently, different results [20]. Given the chronic aspect and stages of glaucoma severity, it is also important to consider heterogeneity in choices within the population.

The design and analysis of stated-preference methods are complex, so it is crucial to follow the steps of the ISPOR Checklist for Conjoint Analysis [10]. Overall, the quality of the included studies was good, but our review identified some methodological shortcomings concerning the current published stated-preference applications in glaucoma management. Most studies reported using appropriate methods to select attributes and levels, but they tended to underreport these aspects, and the use of qualitative methods was limited. Involving patients in attribute development is vital to avoid missing important aspects of decision-making. This aspect could be improved by referring to the guidelines to conduct and report qualitative research for quantitative studies published recently by Hollin et al. [37].

Furthermore, the recruitment of participants was underreported in the majority of studies. High non-response could be due to problems with accessing the survey itself, and herewith lead to non-response bias and limit external validity and generalizability. However, it can be inherently challenging to collect information on non-responders. In addition, the reporting of design features in the reviewed studies was variable, and the rationale behind decisions was often scarce. Several studies also failed to report on their sample size estimation. Therefore, it was difficult to assess whether the studies had recruited an appropriate number of participants for a reliable statistical analysis. Sample-size calculations are particularly challenging for stated-preference studies in health care, and guidance to determine the minimum sample size required to detect differences in preferences has only been available recently [38].

Another point to note is that none of the studies presented an opt-out option in the choice experiment, and therewith, for example, the choice of no treatment. At the same time, it is worth remembering that the option not to choose is often considered less desirable because it means losing information about preferences and running the risk that some people opt-out simply to avoid making difficult choices [39]. Furthermore, it may have serious implications for the experimental design. Pilot testing or tests to measure the level of burden were limited in the included studies. Due to the complexity of preference experiments, pretesting is recommended, and methods for assessing the quality of responses should ideally be incorporated into the instrument design.

To our knowledge, we provide the first systematic review investigating preferences for glaucoma management. The findings of this review could have implications for policy and clinical decision-making. Clinicians should be aware that the effectiveness of glaucoma management is highly valued by patients, and hence, communication at a clinical level must stress its effectiveness. Results of this study could also be useful for policy decision-makers, for example, when considering information on patient preferences in health care decisions. Moreover, this review provides a comprehensive synthesis of available evidence regarding stated-preferences studies investigating glaucoma management and insight into its current limitations. It could, therefore, be helpful for further research.

Several limitations should be considered. First, the external validity of this review is limited by the limitations in the methodology or reporting of the included studies. Second, the use of other search terms could have led to different search results, and non-English articles and conference abstracts were excluded. However, we chose a broad search strategy to identify all relevant articles. Third, the synthesis of preferences included in this review must be considered in the context of heterogeneity of the included attributes and levels. Relative importance highly depends on the range of levels chosen [20]. Meta-analysis and direct comparison of the model parameter estimates were therefore impossible. Finally, this systematic review was not logged in a registry of systematic reviews.

Future research comparing the preferences and priorities of glaucoma patients at different disease severity stages and treatment experiences, such as surgery, could offer insight into how previous experiences affect decision-making. Even though the effectiveness of treatment is and should be the primary focus when patients start treatment, other aspects of care are an understudied area. More research into the preference assessment from the viewpoint of the healthcare provider is necessary to compare their preferences with the patients.

## Conclusion

This review gives an overview of the content and methodology used in studies that measure preferences in the field of glaucoma. Studies were conducted in three applications: treatment, health status valuation, and services. In each type of application, respondents are willing to trade-off, which makes stated-preference techniques suitable. The most frequently included attribute category was outcome, followed by process, and cost. Various attributes and levels for each category (outcome, process, and cost) were included, leading to heterogeneous results in this review. Even though there was variability regarding importance, it does appear that outcome attributes, and specifically effectiveness, often factored heavily in trade-offs relative to other attributes.

### Supplementary information

Appendix I

Appendix II

Appendix III

Appendix IV
