# Supplementary material for: Eliciting preferences in glaucoma management—a systematic review of stated-preference studies
Source: Eye (Lond). 2023 Mar 21;37(15):3137–44. doi: 10.1038/s41433-023-02482-3 (PMC10564796; doi:10.1038/s41433-023-02482-3)
Supplement: Supplementary file 1 — Appendix I [file 41433_2023_2482_MOESM1_ESM.docx]

**APPENDIX I.** Overview of search terms.

| Glaucoma | “glaucoma” OR “glaucoma surgery” |  |
| --- | --- | --- |
| Stated preferences | \| ((((((((((((((conjoint) OR ("conjoint analysis")) OR ("conjoint measurement")) OR ("conjoint studies")) OR ("conjoint choice experiment")) OR ("part-worth utilities")) OR ("functional measurement")) OR ("paired comparisons")) OR ("pairwise choices")) OR ("discrete choice experiment")) OR (dce)) OR ("discrete choice modelling")) OR ("discrete choice modelling")) OR ("discrete choice conjoint experiment")) OR ("stated preference") \| \| --- \| | According to (1, 2) |
| Patient satisfaction/preference | “preference” AND “satisfaction” |  |

**REFERENCES**

1. Clark MD, Determann D, Petrou S, Moro D, de Bekker-Grob EW. Discrete Choice Experiments in Health Economics: A Review of the Literature. PharmacoEconomics. 2014;32(9):883-902.

2. Soekhai V, de Bekker-Grob EW, Ellis AR, Vass CM. Discrete choice experiments in health economics: past, present and future. Pharmacoeconomics. 2019;37(2):201-26.
