# Supplementary material for: Eliciting preferences in glaucoma management—a systematic review of stated-preference studies
Source: Eye (Lond). 2023 Mar 21;37(15):3137–44. doi: 10.1038/s41433-023-02482-3 (PMC10564796; doi:10.1038/s41433-023-02482-3)
Supplement: Supplementary file 2 — Appendix II [file 41433_2023_2482_MOESM2_ESM.docx]

**APPENDIX II.** Overview and data extraction of the included studies.

| **Study** | **Aspinall et al. (2005)** | **Aspinall et al. (2008)** | **Bhargava et al. (2006)** | **Bhargava et al. (2008)** | **Burr et al. (2007)** | **Fenwick et al. (2021** | **Le et al. (2019)** | **Lu et al. (2019)** | **Muth et al. (2021)** | **Muth et al. (2021)** | | **Ozdemir et al. (2017)** | |
| --- | --- | --- | --- | --- | --- | --- | --- | --- | --- | --- | --- | --- | --- |
| General characteristics | | | | | | | | | | | | | |
| Country | UK | UK | UK | UK | UK | Singapore | USA | Australia | Germany | Germany | | | Singapore |
| Study’s objective | To assess the perceived importance of visual field loss on vision-related quality of life for a group of patients with glaucoma. | To investigate the quality of life and priorities of patients with glaucoma. | To determine by conjoint analysis which factors in the management and treatment of glaucoma were of most importance to patients and to relate these factors to the patient's clinical glaucoma condition. | To determine patients’ preferences for provision of glaucoma follow-up services examining preferences for location, access and personnel for delivery of this care. | To estimate a utility-based glaucoma health outcome measure, known as the Glaucoma Utility Index. | To develop and validate the preference-based Glaucoma Utility Instrument (Glau-U) and to ascertain the association between Glau-U utilities and severity of glaucoma and vision impairment. | To quantify patients’ preferences for glaucoma outcomes and use this information to prioritize outcomes that are important to patients. | To explore patient choice in relation to the various attributes of glaucoma outpatient services, the relative importance of different attributes of the service and the trade-offs patients are prepared to make. | To structurally determine patients’ and physicians’ preferences for glaucoma diagnostic methods in order to improve glaucoma patient care and improve patient compliance with follow-up visits. | To structurally determine patients’ and physicians’ preferences for glaucoma diagnostic methods in order to improve glaucoma patient care and improve patient compliance with follow-up visits. | | | To estimate uptake for a technology that delivers sustained-release glaucoma medication and to investigate how uptake varies by product attributes, physician recommendations, peer adoption, and patient characteristics. |
| Affiliation | Visual Impairment Research Group, School of the Built  Environment, Heriot-Watt University, and the Princess Alexandra Eye Pavilion, Lothian Health Board, Edinburgh, the Dep. of Ophthalmology, University of Aberdeen, Scotland; Dep. of ophthalmology, university of Dublin, Dublin, Ireland; Adelphi Group Ltd., Bollington, Allergan Ltd, High Wycombe, United Kingdom | Visual Impairment Research Group, School of the Built  Environment, Heriot-Watt University, and the Princess Alexandra Eye Pavilion, Edinburgh, Scotland; the Department of Ophthalmology, University of Aberdeen, Scotland; and Adelphi Group Ltd., Bollington, United Kingdom | Department of Ophthalmology, Queen’s Medical Centre and the Division of Primary Care, University of Nottingham, Nottingham, United Kingdom | Department of Ophthalmology, Queen’s Medical Centre, Nottingham, United Kingdom. | Health Services Research Unit, University of Aberdeen, Health Economics Research Unit, Institute of Applied Health Sciences, University of Aberdeen, and Department of Ophthalmology, Aberdeen Royal Infirmary, Aberdeen, United Kingdom, Department of ophthalmology, Dublin | Singapore Eye Research Institute, Singapore National Eye Centre, Singapore; Duke-NUS Medical School, National University of Singapore, Singapore; Yong Loo Lin School of Medicine, National University of Singapore, Singapore; Emory University, School of Medicine, Atlanta, Georgia; National University Health System, Singapore | Combined:  Several dep. in ophthalmology, epidemiology and health policy management in the USA | The George Institute for Global Health, UNSW Sydney, Emergency Department, St George Hospital, Faculty of Medicine and Health, University of Sydney, Ophthalmology Department, Westmead Hospital, Sydney, New South Wales, Australia | Department of Ophthalmology, University Hospital, LMU Munich, Munich, Germany | Department of Ophthalmology, University Hospital, LMU Munich, Munich, Germany | | | Health Services and Systems Research Programme, Duke-NUS Medical School; Glaucoma Department, Singapore National Eye Centre, Singapore; School of Medicine, Duke University, Durham, NC, USA |
| Funding | EPSRC for funding support to develop the quality of life questionnaire and Allergan Ltd for funding support for the conjoint analysis study | Supported by an EPSRC grant and by Allergan Europe | None declared | None declared | The study was funded by the WH Ross Foundation for prevention of blindness (Scotland). The Health Services Research Unit and the Health Economics Research Unit are supported by a core grant from the Chief Scientist Office of the Scottish Executive Health Department | Singapore National eye Centre Health Research Endowment fund, Health Services Research Grant. The funders had no role in the design and conduct of the study. | Center of Excellence in Regulatory Science and innovation | None declared | Open Access funding enabled and organized by Projekt DEAL | Open Access funding enabled and organized by Projekt DEAL | | | By a contract between Peregrine Ophthalmic Pte Ltd and Dr EAF as a private consultant |
| Target Population | Glaucoma patients | Glaucoma patients without other ocular comorbidity | Glaucoma patients | Glaucoma patients | Glaucoma patients on treatment | Glaucoma patients | Glaucoma patients  seeking treatment | Glaucoma patients | Ophthalmologists | Glaucoma patients | | | Glaucoma patient, self-reported, topical administration of eye drops |
| Application | Glaucoma-related health state valuation | Glaucoma-related health state valuation | Treatment | Glaucoma service | Glaucoma-related health state valuation | Glaucoma-related health state valuation | Treatment | Glaucoma service | Glaucoma service | Glaucoma service | | | Treatment |
| Generalizability | OK, only open-angle glaucoma, visual acuity of 20/40 or better, without comorbidity | OK, all glaucoma types without comorbidity | OK | OK, all glaucoma patients | OK, all glaucoma types on treatment | OK, no other large ocular comorbidities | OK, mild to moderate open-angle glaucoma | OK, all glaucoma or glaucoma suspect patients, no exclusion criteria | OK, medical studies or medical degree working at the eye hospital | OK, all glaucoma patients treated with medical or surgical therapy | | | OK |
| DCE Characteristics | | | | | | | | | | | | | |
| Attribute and Level Identification | Literature, previous work | Literature, previous work | Expert group | Expert group, patient interviews | Literature, expert opinion, focus group | Literature, focus groups with experts and interviews with patients | Expert focus group, patient focus group, literature review | Literature study, expert group, qualitative research (semi-structured interviews), pilot test | Expert group | Expert group | | | Cognitive interviews with a convenience sample and discussions with experts |
| Attribute and Level selection | Literature, previous work (questionnaire) | Level selection based on literature | Pilot testing with patient interviews | Expert opinion, patient interviews, pilot study | Focus groups, literature research, expert opinion | Literature, focus groups with experts and patients, pretesting | Cognitive interviews, literature, focus groups, expert | Literature, expert opinion, pilot test | Expert group | Expert group | | | Cognitive interviews with a convenience sample and discussions with experts |
| DCE Design | Orthogonal fractional of full factorial design | Fractional factorial design (orthogonal) | Factor design (orthogonal array) | Fractioned factorial design, orthogonal array | Fractional factorial design, fold over techniques to design choice sets | D-optimal experimental design, full profile. | Balanced incomplete block design | Forced-choice design, maximal efficient survey design | Fractioned factorial design, orthogonal array | Fractioned factorial design, orthogonal array | | | Experimental design, 3 blocks |
| No. of profiles | 15 | 15 | 48 | 8 | 32 | 60 (divided into 6 blocks) | 13 | 12 | 32 + 4 holdout profiles | 32 + 4 holdout profiles | | | 24 |
| Choice sets per respondent | 15 | 15 | 8+2 validity test | 8+ 2 holdout | 32 | 10 reduced to 5-6 | 13 | 12 | 36 | 36 | | | 8 |
| Options per choice set | 2 | 2 | Ranking of 10 options | Ranking of 10 options | 2 | 2 | BW scaling | 2 | 5, score version | 5, score version | | | 2 |
| Data collection | | | | | | | | | | | | | |
| Sample size | 108 | 72 | 82 | 100 | 293 | 304 | 274 | 98 | 32 | 41 | | | 500 |
| Mode of Survey | Interviewer administered questionnaire | Interviewer administered questionnaire | Face- to face interview | Face- to face interview | Self-administered questionnaire | Interviewer administered | Online or paper survey | Face-to-face survey | Combination interviewer administered/ self-administered | Combination interviewer administered/self-administered | | | Online survey |
| Ethical Considerations | Ethical approval was granted from the Medical and Clinical Oncology Research Ethics Sub-committee of the Lothian Health Board | Ethical approval was obtained from the local ethics committee | Ethical approval was obtained | Ethic committee approval was granted by the Nottingham Ethics Committee; all patients were supplied with an information sheet and signed a consent form prior to participation in the study | Ethical approval was obtained from the Central Office of Research Ethics Committees | Ethical approval from the SingHealth Centralised Institutional Review Board. All participants provided written informed consent | Ethical approval and informed consent was obtained | Approval was obtained from the South-Eastern Sydney Local Health District and the Western Sydney Local Health District Human Research Ethics Committees | Approval was obtained from the institutional review board of the university eye hospital of the Ludwig-Maximilians-University, Munich, Germany. Informed consent was obtained of each voluntary participant | Approval was obtained from the institutional review board of the university eye hospital of the Ludwig-Maximilians-University, Munich, Germany. Informed consent was obtained of each voluntary participant | | | Ethically reviewed and approved by the National University of Singapore's Institutional Review Board and a waiver of consent was given |
| Attributes and relative Importance | | | | | | | | | | | | | |
| Number of attributes | 5 | 5 | 5 | 5 | 6 | 6 + survival | 13 | 5 | 7 | | 7 | | 4 |
| Levels | 3 per attribute | 3 per attribute | 2-3 per attribute | 2-3 per attribute | 4 per attribute | 3 per attribute | 2 per attribute | 2-4 per attribute | 2-5 per attribute | | 2-5 per attribute | | 2-4 per attribute |
| Relative importance reported | yes | yes | yes | yes | no | no | yes | no | yes | | yes | | no |
| Most important attribute | Central and near vision | Central and near vision | Risk of being unable to drive | Travel time | Central and near vision | Movement | Having adequate IOP control | Senior clinician | Sensitivity of the examination method | | Sensitivity of the examination method | | Frequency |
| All attributes | Central and near vision, darkness and glare, outdoor mobility, household chores, peripheral vision | Central and near vision, outdoor mobility, bumping into things/peripheral vision, household chores, problems with darkness and glare from bright lights | Risk of being unable to drive, risk of blindness, preference for trabeculectomy, preference for topical therapy, risk of early visual loss | Travel time, level of healthcare professional, no. of visits, access, wait in clinic | Central and near vision, lighting and glare, mobility, activities of daily living, eye discomfort, other effects of glaucoma | Activities of daily living, lighting and glare, movement, eye discomfort, other effects of glaucoma and its treatment, social and emotional effects of glaucoma, duration in this health state | Adequate IOP control, maintain mobility outside the home, depth perception, drive a car during the day, peripheral vision, maintain mobility inside the home, see in very dim or very bright light, drive a car at night, read fine print, no ocular surface symptoms, distinguish colour, reduce number or IOP lowering drops, maintain appearance of the eye | Cost, wait time, continuity, expertise, location | Examination comfort, frequency how often the examination needs to be performed, follow-up examination necessary in case of suspicious result in order to confirm result, cost for the patient, travel time to examination site, sensitivity and specificity of the examination method | | Examination comfort, frequency how often the examination needs to be performed, follow-up examination necessary in case of suspicious result in order to confirm result, cost for the patient, travel time to examination site, sensitivity and specificity of the examination method | | Interval between administrations, annual out-of-pocket cost, physician's recommendation, and perceived adoption rate defined as percentage of patients seen in the clinic who adopted the new technology |
