# Supplementary material for: Eliciting preferences in glaucoma management—a systematic review of stated-preference studies
Source: Eye (Lond). 2023 Mar 21;37(15):3137–44. doi: 10.1038/s41433-023-02482-3 (PMC10564796; doi:10.1038/s41433-023-02482-3)
Supplement: Supplementary file 3 — Appendix III [file 41433_2023_2482_MOESM3_ESM.docx]

**APPENDIX III.** Overview of included attributes and their levels.

| Category | | Attribute | Levels | Study |
| --- | --- | --- | --- | --- |
| Outcome | Effectiveness | Risk of being unable to drive | Moderate visual loss, defined as being ineligible to hold a U.K. driver’s license (5%, 10%, or 20%) at 10 years. | Bhargava (2006) |
|  |  | Risk of blindness | (of 2% or 5%) at 10 years. | Bhargava (2006) |
|  |  | Have control of intraocular pressure | BWS | Le (2019) |
|  |  | Reduce number of IOP lowering drops | BWS | Le (2019) |
|  |  | Survival | (duration in this health state) with 4 possible levels (5, 10, 15, and 20 years) | Fenwick (2021) |
|  |  | Sensitivity | 40%; 70%; 90% | Muth (2021) |
|  |  | Specificity | 50%; 80%; 90% | Muth (2021) |
|  |  | Sensitivity | 40%; 70%; 90% | Muth (2021) (physician) |
|  |  | Specificity | 50%; 80%; 90% | Muth (2021) (physician) |
|  | Adverse effects | Risk of early visual loss | small [defined as <1%] increased risk versus no increased risk | Bhargava (2006) |
|  |  | No ocular surface symptoms | BWS | Le (2019) |
|  |  | Maintain appearance of the eye (cosmesis) | BWS | Le (2019) |
|  |  | Eye discomfort | Never, sometimes, or often | Fenwick (2021) |
|  |  | Other effects of glaucoma and its treatment | Never, sometimes, or often | Fenwick (2021) |
|  |  | Eye discomfort | no difficulty, some difficulty, quite a lot of difficulty or severe difficulty | Burr (2007) |
|  |  | Other effects | no difficulty, some difficulty, quite a lot of difficulty or severe difficulty | Burr (2007) |
|  | Quality of life | Drive a car during the day | BWS | Le (2019) |
|  |  | Maintain mobility outside the home | BWS | Le (2019) |
|  |  | Maintain mobility inside the home | BWS | Le (2019) |
|  |  | Perceive depth | BWS | Le (2019) |
|  |  | Drive a car at night | BWS | Le (2019) |
|  |  | Read fine print | BWS | Le (2019) |
|  |  | See in very dim or very bright light | BWS | Le (2019) |
|  |  | See things off to the side (peripheral vision) | BWS | Le (2019) |
|  |  | Distinguish colour | BWS | Le (2019) |
|  |  | Activities of daily living | no difficulty, some difficulty, or severe difficulty | Fenwick (2021) |
|  |  | Lighting and glare | no difficulty, some difficulty, or severe difficulty | Fenwick (2021) |
|  |  | Movement | no difficulty, some difficulty, or severe difficulty | Fenwick (2021) |
|  |  | Social and emotional effects | Never, sometimes, or often | Fenwick (2021) |
|  |  | Central near vision | no difficulty, some difficulty, quite a lot of difficulty or severe difficulty | Burr (2007) |
|  |  | Lighting and glare | no difficulty, some difficulty, quite a lot of difficulty or severe difficulty | Burr (2007) |
|  |  | Mobility | no difficulty, some difficulty, quite a lot of difficulty or severe difficulty | Burr (2007) |
|  |  | Activities of daily living | no difficulty, some difficulty, quite a lot of difficulty or severe difficulty | Burr (2007) |
|  |  | Reading or seeing details | No problems, a few problems, a lot of problems | Aspinall (2008) |
|  |  | Getting about outside the house | No problems, a few problems, a lot of problems | Aspinall (2008) |
|  |  | Darkness or glare | No problems, a few problems, a lot of problems | Aspinall (2008) |
|  |  | Bumping into and seeing objects | No problems, a few problems, a lot of problems | Aspinall (2008) |
|  |  | Household chores | No problems, a few problems, a lot of problems | Aspinall (2008) |
|  |  | Central vision | ‘no difficulties,’ ‘a few difficulties,’ or ‘a lot of difficulties’ | Aspinall (2005) |
|  |  | Darkness and glare | ‘no difficulties,’ ‘a few difficulties,’ or ‘a lot of difficulties’ | Aspinall (2005) |
|  |  | Outdoor mobility | ‘no difficulties,’ ‘a few difficulties,’ or ‘a lot of difficulties’ | Aspinall (2005) |
|  |  | Household chores | ‘no difficulties,’ ‘a few difficulties,’ or ‘a lot of difficulties’ | Aspinall (2005) |
|  |  | Peripheral vision | ‘no difficulties,’ ‘a few difficulties,’ or ‘a lot of difficulties’ | Aspinall (2005) |
| Process | Mode of administration | Preference for trabeculectomy | yes or no | Bhargava (2006) |
|  |  | Preference for topical treatment | yes or no | Bhargava (2006) |
|  |  | Adoption rate among peers | 5, 30, 70% percentage of patient treated in your clinic who adopted the injectable solution | Ozdemir (2017) |
|  |  | Doctor’s recommendation | Injectable solution, eye drops | Ozdemir (2017) |
|  |  | Level of health care professional | optician and doctor | Bhargava (2008) |
|  |  | Continuity | Single doctor, different doctors | Lu (2019) |
|  |  | Expertise | Optometrist, junior eye doctor, senior eye doctor | Lu (2019) |
|  |  | Comfort | Comfortable and quick; (2) slightly uncomfortable and a few minutes; (3) very uncomfortable and 15 min | Muth (2021) |
|  |  | Comfort | Comfortable and quick; (2) slightly uncomfortable and a few minutes; (3) very uncomfortable and 15 min | Muth (2021) (physician) |
|  | Frequency | Interval between injections | Every 4, 6, or 9 months | Ozdemir (2017) |
|  |  | No. of visits | one visit and two visits | Bhargava (2008) |
|  |  | Frequency | no; (2) yes | Muth (2021) |
|  |  | Follow-up | no; (2) yes | Muth (2021) |
|  |  | Frequency | once; (2) every five years; (3) every two years; (4) every year | Muth (2021) (physician) |
|  |  | Follow-up | once; (2) every five years; (3) every two years; (4) every year | Muth (2021) (physician) |
|  | Location | Travel time | 30, 60 and 120 min. | Bhargava (2008) |
|  |  | Access | Easy access was defined, for those on public transport, as requiring no change of bus or tram and that the final stop was less than 20 min from the clinic. For those in cars, easy access was defined as always having access to a driver and that the time to park was less than 20 min. | Bhargava (2008) |
|  |  | Location | Inside large hospital, off-site clinic (e.g., shopping centre) | Lu (2019) |
|  |  | Travel time | < 30 min.; (2) ca. 1 h; (3) ca. 2 h | Muth (2021) |
|  |  | Travel time | < 30 min.; (2) ca. 1 h; (3) ca. 2 h | Muth (2021) (physician) |
|  | Waiting time | Wait in clinic | 15, 60, 120, 240 in min | Bhargava (2008) |
|  |  | Wait time | Level 1 was good and was defined as all tests and the clinic visit completed within 1 h of their scheduled appointment time, and a poor appointment meant leaving at more than 1 h after the appointment time. | Lu (2019) |
| Cost | Cost | Out-of-pocket costs per year | Free, 100, 500 or 1000$ | Ozdemir (2017) |
|  |  | Cost | 0, 100, 250, 400, Australian $ | Lu (2019) |
|  |  | Cost | 10€; (3) 20€; (4) 70€; (5) 140€ | Muth (2021) |
|  |  | Cost | 10€; (3) 20€; (4) 70€; (5) 140€ | Muth (2021) (physician) |
