# Supplementary material for: Eliciting preferences in glaucoma management—a systematic review of stated-preference studies
Source: Eye (Lond). 2023 Mar 21;37(15):3137–44. doi: 10.1038/s41433-023-02482-3 (PMC10564796; doi:10.1038/s41433-023-02482-3)
Supplement: Supplementary file 4 — Appendix IV [file 41433_2023_2482_MOESM4_ESM.docx]

**APPENDIX IV.** Relative importance of attributes per study.

| **Study** |  | **Attribute** |  | **Attribute (sub)category** | |  | **Relative importance** |  | **Score** |
| --- | --- | --- | --- | --- | --- | --- | --- | --- | --- |
|  |  |  |  |  |  |  |  |  |  |
| Aspinall (2005) |  | Central vision |  | Outcome | Quality of life |  | (histogram) |  | 1 |
|  |  | Darkness and glare |  | Outcome | Quality of life |  |  |  | 5 |
|  |  | Outdoor mobility |  | Outcome | Quality of life |  |  |  | 2 |
|  |  | Household chores |  | Outcome | Quality of life |  |  |  | 3 |
|  |  | Peripheral vision |  | Outcome | Quality of life |  |  |  | 4 |
| Aspinall (2008) |  | Reading or seeing details |  | Outcome | Quality of life |  | 32.3% |  | 1 |
|  |  | Getting about outside the house |  | Outcome | Quality of life |  | 25.0% |  | 2 |
|  |  | Darkness or glare |  | Outcome | Quality of life |  | 16.2% |  | 3 |
|  |  | Bumping into and seeing objects |  | Outcome | Quality of life |  | 15.7% |  | 4 |
|  |  | Household chores |  | Outcome | Quality of life |  | 10.9% |  | 5 |
| Bhargava (2006) |  | Risk of being unable to drive |  | Outcome | Effectiveness |  | 38.6% |  | 1 |
|  |  | Risk of blindness |  | Outcome | Effectiveness |  | 26.9% |  | 2 |
|  |  | Preference for trabeculectomy |  | Process | Mode of administration |  | 14.9% |  | 3 |
|  |  | Preference for topical treatment |  | Process | Mode of administration |  | 10.8% |  | 4 |
|  |  | Risk of early visual loss |  | Outcome | Adverse effects |  | 8.9% |  | 5 |
| Bhargava (2008) |  | Travel time |  | Process | Location |  | 35.4% |  | 1 |
|  |  | Level of health care professional |  | Process | Mode of administration |  | 25.9% |  | 2 |
|  |  | No. of visits |  | Process | Frequency |  | 13.8% |  | 3 |
|  |  | Access |  | Process | Location |  | 15.3% |  | 4 |
|  |  | Wait in clinic |  | Process | Waiting time |  | 9.6% |  | 5 |
| Burr (2007) |  | Central near vision |  | Outcome | Quality of life |  | 32.2% |  | 1 |
|  |  | Lighting and glare |  | Outcome | Quality of life |  | 7.0% |  | 4 |
|  |  | Mobility |  | Outcome | Quality of life |  | 23.7% |  | 3 |
|  |  | Activities of daily living |  | Outcome | Quality of life |  | 25.7% |  | 2 |
|  |  | Eye discomfort |  | Outcome | Adverse effects |  | 6.2% |  | 5 |
|  |  | Other effects |  | Outcome | Other |  | 5.2% |  | 6 |
| Fenwick (2021) |  | Activities of daily living |  | Outcome | Quality of life |  | 20.6% |  | 3 |
|  |  | Lighting and glare |  | Outcome | Quality of life |  | 7.9% |  | 5 |
|  |  | Movement |  | Outcome | Quality of life |  | 25.3% |  | 2 |
|  |  | Eye discomfort |  | Outcome | Adverse effects |  | 0.0% |  | 7 |
|  |  | Other effects of glaucoma and its treatment |  | Outcome | Other |  | 3.2% |  | 6 |
|  |  | Social and emotional effects |  | Outcome | Quality of life |  | 15.8% |  | 4 |
|  |  | Survival |  | Outcome | Effectiveness |  | 27.3% |  | 1 |
| Le (2019) |  | Have control of intraocular pressure |  | Outcome | Effectiveness |  | 14.8% |  | 1 |
|  |  | Drive a car during the day |  | Outcome | Quality of life |  | 14.4% |  | 2 |
|  |  | Maintain mobility outside the home |  | Outcome | Quality of life |  | 10.8% |  | 3 |
|  |  | Maintain mobility inside the home |  | Outcome | Quality of life |  | 10.3% |  | 4 |
|  |  | Perceive depth |  | Outcome | Quality of life |  | 9.1% |  | 5 |
|  |  | Drive a car at night |  | Outcome | Quality of life |  | 8.6% |  | 6 |
|  |  | Read fine print |  | Outcome | Quality of life |  | 7.3% |  | 7 |
|  |  | See in very dim or very bright light |  | Outcome | Quality of life |  | 6.6% |  | 8 |
|  |  | No ocular surface symptoms |  | Outcome | Adverse effects |  | 5.8% |  | 9 |
|  |  | See things off to the side (peripheral vision) |  | Outcome | Quality of life |  | 5.1% |  | 10 |
|  |  | Distinguish colour |  | Outcome | Quality of life |  | 4.0% |  | 11 |
|  |  | Reduce number of IOP lowering drops |  | Outcome | Effectiveness |  | 1.8% |  | 12 |
|  |  | Maintain appearance of the eye (cosmesis) |  | Outcome | Adverse effects |  | 1.4% |  | 13 |
| Lu (2019) |  | Cost |  | Costs | Costs |  | 29.1% |  | 2 |
|  |  | Wait time |  | Process | Waiting time |  | 5.8% |  | 4 |
|  |  | Continuity |  | Process | Mode of administration |  | 13.0% |  | 3 |
|  |  | Expertise |  | Process | Mode of administration |  | 48.9% |  | 1 |
|  |  | Location |  | Process | Location |  | 3.2% |  | 5 |
| Muth (2021) |  | Comfort |  | Process | Mode of administration |  | 7.8% |  | 6 |
|  |  | Frequency |  | Process | Frequency |  | 11.7% |  | 5 |
|  |  | Follow-up |  | Process | Frequency |  | 11.9% |  | 4 |
|  |  | Cost |  | Costs | Costs |  | 17.6% |  | 2 |
|  |  | Travel time |  | Process | Location |  | 6.3% |  | 7 |
|  |  | Sensitivity |  | Outcome | Accuracy |  | 31.7% |  | 1 |
|  |  | Specificity |  | Outcome | Accuracy |  | 13.0% |  | 3 |
| Muth (2021) (physician) |  | Comfort |  | Process | Mode of administration |  | 9.9% |  | 4 |
|  |  | Frequency |  | Process | Frequency |  | 6.6% |  | 6 |
|  |  | Follow-up |  | Process | Frequency |  | 5.8% |  | 7 |
|  |  | Cost |  | Costs | Costs |  | 11.2% |  | 3 |
|  |  | Travel time |  | Process | Location |  | 7.1% |  | 5 |
|  |  | Sensitivity |  | Outcome | Accuracy |  | 41.4% |  | 1 |
|  |  | Specificity |  | Outcome | Accuracy |  | 17.9% |  | 2 |
| Ozdemir (2017) |  | Interval between injections |  | Process | Frequency |  | 62.2% |  | 1 |
| For age <65 |  | Out-of-pocket costs per year |  | Costs | Costs |  | 26.7% |  | 2 |
|  |  | Adoption rate among peers |  | Process | Mode of administration |  | 5.0% |  | 4 |
|  |  | Doctor’s recommendation |  | Process | Mode of administration |  | 6.1% |  | 3 |
| Ozdemir (2017) |  | Interval between injections |  | Process | Frequency |  | 63.7% |  | 1 |
| For age ≥65 |  | Out-of-pocket costs per year |  | Costs | Costs |  | 24.9% |  | 2 |
|  |  | Adoption rate among peers |  | Process | Mode of administration |  | 5.1% |  | 4 |
|  |  | Doctor’s recommendation |  | Process | Mode of administration |  | 6.3% |  | 3 |
